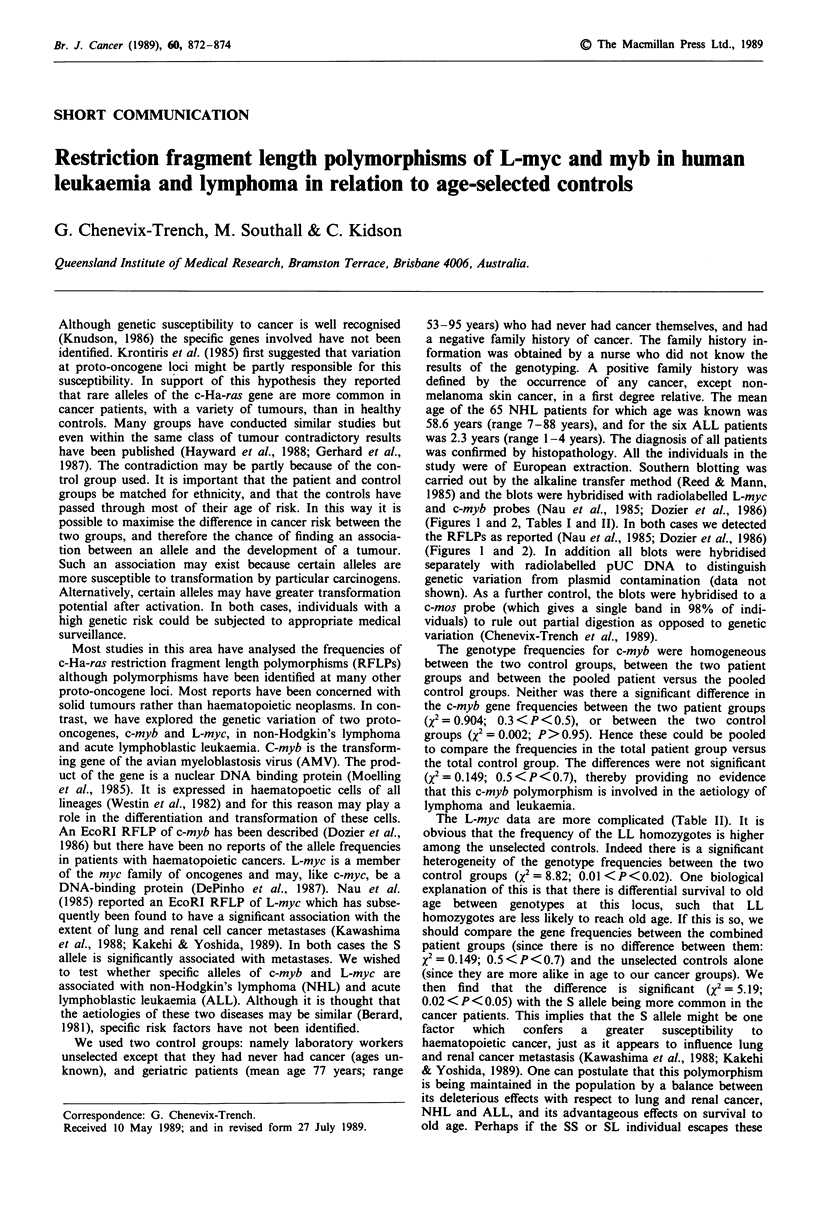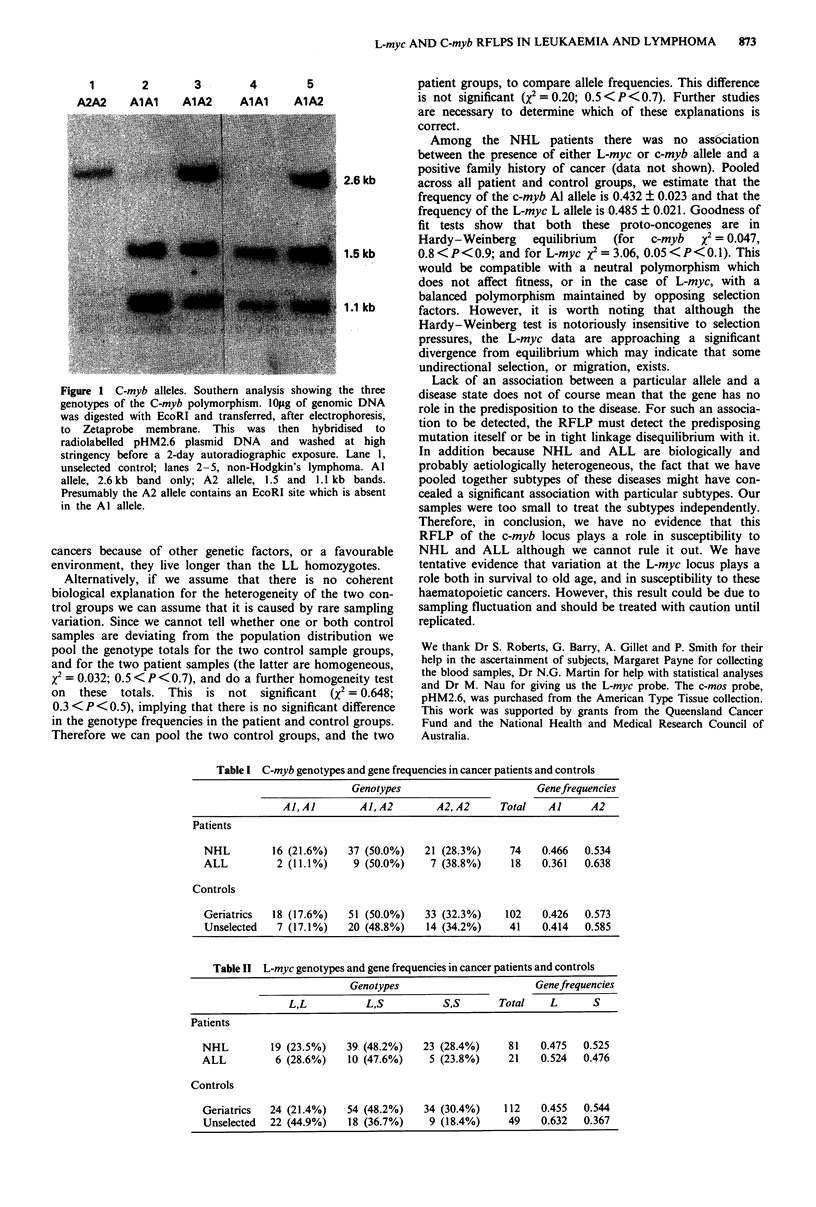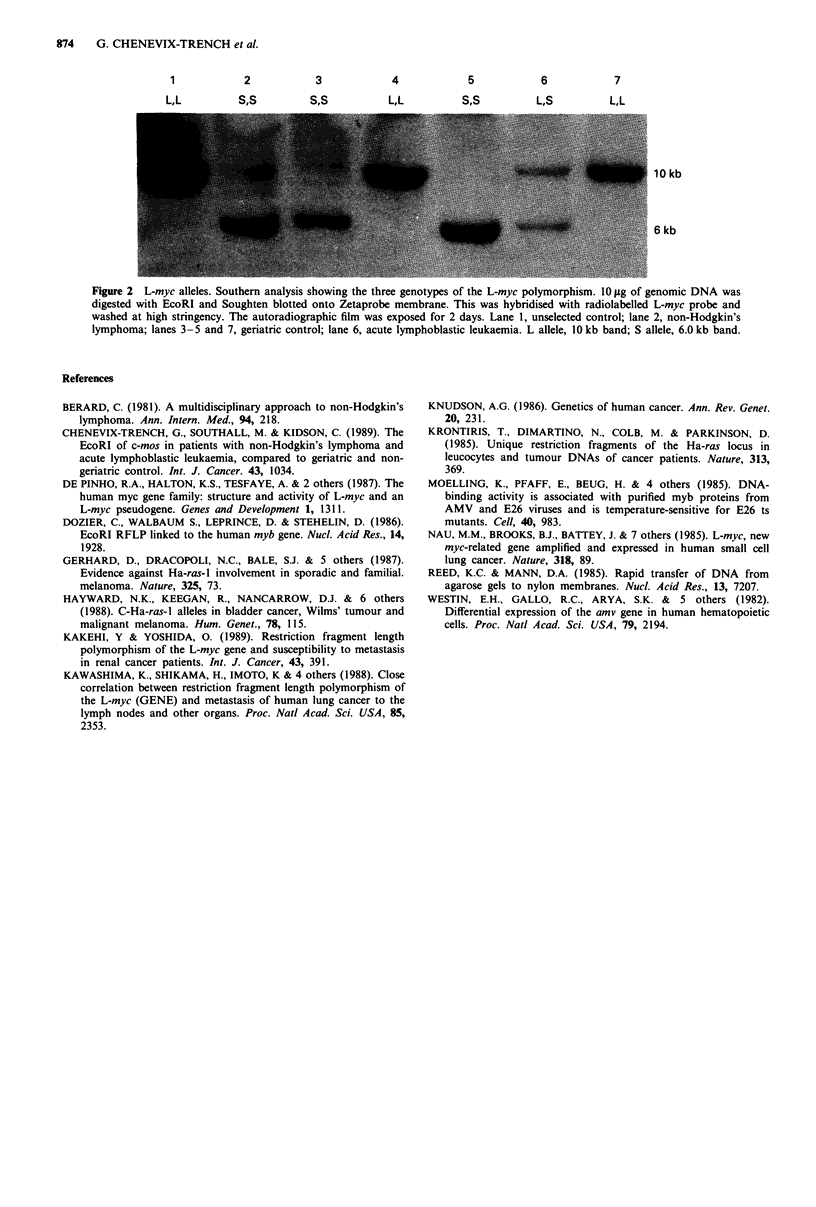# Restriction fragment length polymorphisms of L-myc and myb in human leukaemia and lymphoma in relation to age-selected controls.

**DOI:** 10.1038/bjc.1989.382

**Published:** 1989-12

**Authors:** G. Chenevix-Trench, M. Southall, C. Kidson

**Affiliations:** Queensland Institute of Medical Research, Brisbane, Australia.

## Abstract

**Images:**


Br. J. Cancer (1989), 60, 872–874 © The Macmillan Press Ltd., 1989

SHORT COMMUNICATION

### Restriction fragment length polymorphisms of L-myc and myb in human leukaemia and lymphoma in relation to age-selected controls

G. Chenevix-Trench, M. Southall & C. Kidson

*Queensland Institute of Medical Research, Bramston Terrace, Brisbane 4006, Australia.*

Although genetic susceptibility to cancer is well recognised (Knudson, 1986) the specific genes involved have not been identified. Krontiris *et al.* (1985) first suggested that variation at proto-oncogene loci might be partly responsible for this susceptibility. In support of this hypothesis they reported that rare alleles of the c-Ha-*ras* gene are more common in cancer patients, with a variety of tumours, than in healthy controls. Many groups have conducted similar studies but even within the same class of tumour contradictory results have been published (Hayward *et al.*, 1988; Gerhard *et al.*, 1987). The contradiction may be partly because of the control group used. It is important that the patient and control groups be matched for ethnicity, and that the controls have passed through most of their age of risk. In this way it is possible to maximise the difference in cancer risk between the two groups, and therefore the chance of finding an association between an allele and the development of a tumour. Such an association may exist because certain alleles are more susceptible to transformation by particular carcinogens. Alternatively, certain alleles may have greater transformation potential after activation. In both cases, individuals with a high genetic risk could be subjected to appropriate medical surveillance.

Most studies in this area have analysed the frequencies of c-Ha-*ras* restriction fragment length polymorphisms (RFLPs) although polymorphisms have been identified at many other proto-oncogene loci. Most reports have been concerned with solid tumours rather than haematopoietic neoplasms. In contrast, we have explored the genetic variation of two proto-oncogenes, c-*myb* and L-*myc*, in non-Hodgkin's lymphoma and acute lymphoblastic leukaemia. C-*myb* is the transforming gene of the avian myeloblastosis virus (AMV). The product of the gene is a nuclear DNA binding protein (Moelling *et al.*, 1985). It is expressed in haematopoetic cells of all lineages (Westin *et al.*, 1982) and for this reason may play a role in the differentiation and transformation of these cells. An EcoRI RFLP of c-*myb* has been described (Dozier *et al.*, 1986) but there have been no reports of the allele frequencies in patients with haematopoietic cancers. L-*myc* is a member of the *myc* family of oncogenes and may, like c-*myc*, be a DNA-binding protein (DePinho *et al.*, 1987). Nau *et al.* (1985) reported an EcoRI RFLP of L-*myc* which has subsequently been found to have a significant association with the extent of lung and renal cell cancer metastases (Kawashima *et al.*, 1988; Kakehi & Yoshida, 1989). In both cases the S allele is significantly associated with metastases. We wished to test whether specific alleles of c-*myb* and L-*myc* are associated with non-Hodgkin's lymphoma (NHL) and acute lymphoblastic leukaemia (ALL). Although it is thought that the aetiologies of these two diseases may be similar (Berard, 1981), specific risk factors have not been identified.

We used two control groups: namely laboratory workers unselected except that they had never had cancer (ages unknown), and geriatric patients (mean age 77 years; range 53–95 years) who had never had cancer themselves, and had a negative family history of cancer. The family history information was obtained by a nurse who did not know the results of the genotyping. A positive family history was defined by the occurrence of any cancer, except non-melanoma skin cancer, in a first degree relative. The mean age of the 65 NHL patients for which age was known was 58.6 years (range 7–88 years), and for the six ALL patients was 2.3 years (range 1–4 years). The diagnosis of all patients was confirmed by histopathology. All the individuals in the study were of European extraction. Southern blotting was carried out by the alkaline transfer method (Reed & Mann, 1985) and the blots were hybridised with radiolabelled L-*myc* and c-*myb* probes (Nau *et al.*, 1985; Dozier *et al.*, 1986) (Figures 1 and 2, Tables I and II). In both cases we detected the RFLPs as reported (Nau *et al.*, 1985; Dozier *et al.*, 1986) (Figures 1 and 2). In addition all blots were hybridised separately with radiolabelled pUC DNA to distinguish genetic variation from plasmid contamination (data not shown). As a further control, the blots were hybridised to a c-*mos* probe (which gives a single band in 98% of individuals) to rule out partial digestion as opposed to genetic variation (Chenevix-Trench *et al.*, 1989).

The genotype frequencies for c-*myb* were homogeneous between the two control groups, between the two patient groups and between the pooled patient versus the pooled control groups. Neither was there a significant difference in the c-*myb* gene frequencies between the two patient groups ($\chi^2 = 0.904$; $0.3 < P < 0.5$), or between the two control groups ($\chi^2 = 0.002$; $P > 0.95$). Hence these could be pooled to compare the frequencies in the total patient group versus the total control group. The differences were not significant ($\chi^2 = 0.149$; $0.5 < P < 0.7$), thereby providing no evidence that this c-*myb* polymorphism is involved in the aetiology of lymphoma and leukaemia.

The L-*myc* data are more complicated (Table II). It is obvious that the frequency of the LL homozygotes is higher among the unselected controls. Indeed there is a significant heterogeneity of the genotype frequencies between the two control groups ($\chi^2 = 8.82$; $0.01 < P < 0.02$). One biological explanation of this is that there is differential survival to old age between genotypes at this locus, such that LL homozygotes are less likely to reach old age. If this is so, we should compare the gene frequencies between the combined patient groups (since there is no difference between them: $\chi^2 = 0.149$; $0.5 < P < 0.7$) and the unselected controls alone (since they are more alike in age to our cancer groups). We then find that the difference is significant ($\chi^2 = 5.19$; $0.02 < P < 0.05$) with the S allele being more common in the cancer patients. This implies that the S allele might be one factor which confers a greater susceptibility to haematopoietic cancer, just as it appears to influence lung and renal cancer metastasis (Kawashima *et al.*, 1988; Kakehi & Yoshida, 1989). One can postulate that this polymorphism is being maintained in the population by a balance between its deleterious effects with respect to lung and renal cancer, NHL and ALL, and its advantageous effects on survival to old age. Perhaps if the SS or SL individual escapes these

Correspondence: G. Chenevix-Trench.
Received 10 May 1989; and in revised form 27 July 1989.

**Figure 1** C-*myb* alleles. Southern analysis showing the three genotypes of the C-*myb* polymorphism. 10μg of genomic DNA was digested with EcoRI and transferred, after electrophoresis, to Zetaprobe membrane. This was then hybridised to radiolabelled pHM2.6 plasmid DNA and washed at high stringency before a 2-day autoradiographic exposure. Lane 1, unselected control; lanes 2–5, non-Hodgkin's lymphoma. A1 allele, 2.6 kb band only; A2 allele, 1.5 and 1.1 kb bands. Presumably the A2 allele contains an EcoRI site which is absent in the A1 allele.

cancers because of other genetic factors, or a favourable environment, they live longer than the LL homozygotes.

Alternatively, if we assume that there is no coherent biological explanation for the heterogeneity of the two control groups we can assume that it is caused by rare sampling variation. Since we cannot tell whether one or both control samples are deviating from the population distribution we pool the genotype totals for the two control sample groups, and for the two patient samples (the latter are homogeneous, $\chi^2 = 0.032$; $0.5 < P < 0.7$), and do a further homogeneity test on these totals. This is not significant ($\chi^2 = 0.648$; $0.3 < P < 0.5$), implying that there is no significant difference in the genotype frequencies in the patient and control groups. Therefore we can pool the two control groups, and the two patient groups, to compare allele frequencies. This difference is not significant ($\chi^2 = 0.20$; $0.5 < P < 0.7$). Further studies are necessary to determine which of these explanations is correct.

Among the NHL patients there was no association between the presence of either L-*myc* or c-*myb* allele and a positive family history of cancer (data not shown). Pooled across all patient and control groups, we estimate that the frequency of the c-*myb* Al allele is 0.432 ± 0.023 and that the frequency of the L-*myc* L allele is 0.485 ± 0.021. Goodness of fit tests show that both these proto-oncogenes are in Hardy–Weinberg equilibrium (for c-*myb* $\chi^2 = 0.047$, $0.8 < P < 0.9$; and for L-*myc* $\chi^2 = 3.06$, $0.05 < P < 0.1$). This would be compatible with a neutral polymorphism which does not affect fitness, or in the case of L-*myc*, with a balanced polymorphism maintained by opposing selection factors. However, it is worth noting that although the Hardy–Weinberg test is notoriously insensitive to selection pressures, the L-*myc* data are approaching a significant divergence from equilibrium which may indicate that some undirectional selection, or migration, exists.

Lack of an association between a particular allele and a disease state does not of course mean that the gene has no role in the predisposition to the disease. For such an association to be detected, the RFLP must detect the predisposing mutation iteself or be in tight linkage disequilibrium with it. In addition because NHL and ALL are biologically and probably aetiologically heterogeneous, the fact that we have pooled together subtypes of these diseases might have concealed a significant association with particular subtypes. Our samples were too small to treat the subtypes independently. Therefore, in conclusion, we have no evidence that this RFLP of the c-*myb* locus plays a role in susceptibility to NHL and ALL although we cannot rule it out. We have tentative evidence that variation at the L-*myc* locus plays a role both in survival to old age, and in susceptibility to these haematopoietic cancers. However, this result could be due to sampling fluctuation and should be treated with caution until replicated.

We thank Dr S. Roberts, G. Barry, A. Gillet and P. Smith for their help in the ascertainment of subjects, Margaret Payne for collecting the blood samples, Dr N.G. Martin for help with statistical analyses and Dr M. Nau for giving us the L-*myc* probe. The c-*mos* probe, pHM2.6, was purchased from the American Type Tissue collection. This work was supported by grants from the Queensland Cancer Fund and the National Health and Medical Research Council of Australia.

**Table I** C-*myb* genotypes and gene frequencies in cancer patients and controls

| | *Genotypes* | | | | *Gene frequencies* | |
|---|---|---|---|---|---|---|
| | *A1, A1* | *A1, A2* | *A2, A2* | *Total* | *A1* | *A2* |
| Patients | | | | | | |
| NHL | 16 (21.6%) | 37 (50.0%) | 21 (28.3%) | 74 | 0.466 | 0.534 |
| ALL | 2 (11.1%) | 9 (50.0%) | 7 (38.8%) | 18 | 0.361 | 0.638 |
| Controls | | | | | | |
| Geriatrics | 18 (17.6%) | 51 (50.0%) | 33 (32.3%) | 102 | 0.426 | 0.573 |
| Unselected | 7 (17.1%) | 20 (48.8%) | 14 (34.2%) | 41 | 0.414 | 0.585 |

**Table II** L-*myc* genotypes and gene frequencies in cancer patients and controls

| | *Genotypes* | | | | *Gene frequencies* | |
|---|---|---|---|---|---|---|
| | *L,L* | *L,S* | *S,S* | *Total* | *L* | *S* |
| Patients | | | | | | |
| NHL | 19 (23.5%) | 39 (48.2%) | 23 (28.4%) | 81 | 0.475 | 0.525 |
| ALL | 6 (28.6%) | 10 (47.6%) | 5 (23.8%) | 21 | 0.524 | 0.476 |
| Controls | | | | | | |
| Geriatrics | 24 (21.4%) | 54 (48.2%) | 34 (30.4%) | 112 | 0.455 | 0.544 |
| Unselected | 22 (44.9%) | 18 (36.7%) | 9 (18.4%) | 49 | 0.632 | 0.367 |

**Figure 2** L-*myc* alleles. Southern analysis showing the three genotypes of the L-*myc* polymorphism. 10 µg of genomic DNA was digested with EcoRI and Soughten blotted onto Zetaprobe membrane. This was hybridised with radiolabelled L-*myc* probe and washed at high stringency. The autoradiographic film was exposed for 2 days. Lane 1, unselected control; lane 2, non-Hodgkin's lymphoma; lanes 3–5 and 7, geriatric control; lane 6, acute lymphoblastic leukaemia. L allele, 10 kb band; S allele, 6.0 kb band.

### References

BERARD, C. (1981). A multidisciplinary approach to non-Hodgkin's lymphoma. *Ann. Intern. Med.*, **94**, 218.

CHENEVIX-TRENCH, G., SOUTHALL, M. & KIDSON, C. (1989). The EcoRI of c-*mos* in patients with non-Hodgkin's lymphoma and acute lymphoblastic leukaemia, compared to geriatric and non-geriatric control. *Int. J. Cancer*. **43**, 1034.

DE PINHO, R.A., HALTON, K.S., TESFAYE, A. & 2 others (1987). The human myc gene family: structure and activity of L-*myc* and an L-*myc* pseudogene. *Genes and Development* **1**, 1311.

DOZIER, C., WALBAUM S., LEPRINCE, D. & STEHELIN, D. (1986). EcoRI RFLP linked to the human *myb* gene. *Nucl. Acid Res.*, **14**, 1928.

GERHARD, D., DRACOPOLI, N.C., BALE, S.J. & 5 others (1987). Evidence against Ha-*ras*-1 involvement in sporadic and familial. melanoma. *Nature*, **325**, 73.

HAYWARD, N.K., KEEGAN, R., NANCARROW, D.J. & 6 others (1988). C-Ha-*ras*-1 alleles in bladder cancer, Wilms' tumour and malignant melanoma. *Hum. Genet.*, **78**, 115.

KAKEHI, Y & YOSHIDA, O. (1989). Restriction fragment length polymorphism of the L-*myc* gene and susceptibility to metastasis in renal cancer patients. *Int. J. Cancer*, **43**, 391.

KAWASHIMA, K., SHIKAMA, H., IMOTO, K & 4 others (1988). Close correlation between restriction fragment length polymorphism of the L-*myc* (GENE) and metastasis of human lung cancer to the lymph nodes and other organs. *Proc. Natl Acad. Sci. USA*, **85**, 2353.

KNUDSON, A.G. (1986). Genetics of human cancer. *Ann. Rev. Genet.* **20**, 231.

KRONTIRIS, T., DIMARTINO, N., COLB, M. & PARKINSON, D. (1985). Unique restriction fragments of the Ha-*ras* locus in leucocytes and tumour DNAs of cancer patients. *Nature*, **313**, 369.

MOELLING, K., PFAFF, E., BEUG, H. & 4 others (1985). DNA-binding activity is associated with purified myb proteins from AMV and E26 viruses and is temperature-sensitive for E26 ts mutants. *Cell*, **40**, 983.

NAU, M.M., BROOKS, B.J., BATTEY, J. & 7 others (1985). L-*myc*, new *myc*-related gene amplified and expressed in human small cell lung cancer. *Nature*, **318**, 89.

REED, K.C. & MANN, D.A. (1985). Rapid transfer of DNA from agarose gels to nylon membranes. *Nucl. Acid Res.*, **13**, 7207.

WESTIN, E.H., GALLO, R.C., ARYA, S.K. & 5 others (1982). Differential expression of the *amv* gene in human hematopoietic cells. *Proc. Natl Acad. Sci. USA*, **79**, 2194.